# Effects of Post-UV-Curing on the Flexural and Absorptive Behaviour of FDM-3D-Printed Poly(lactic acid) Parts

**DOI:** 10.3390/polym15020348

**Published:** 2023-01-09

**Authors:** Tarkan Akderya

**Affiliations:** Department of Biomedical Engineering, Faculty of Engineering and Architecture, University of Bakırçay, 35665 Izmir, Turkey; tarkan.akderya@bakircay.edu.tr

**Keywords:** fused deposition modelling, post-UV-curing, poly(lactic acid), flexural properties, finite element analysis

## Abstract

In this study, the effects of the post-ultraviolet-curing process on the flexural, absorptive, and morphological properties of poly(lactic acid) specimens produced using a fused deposition modelling technique 3D printer were experimentally investigated. In this direction, 15, 30, 45, and 60 min post-UV-curing processes were applied to poly(lactic acid) three-point bending and absorption specimens produced at 190 and 200 °C. Three-point bending tests and morphological analyses were applied after the post-ultraviolet-curing process, and absorption tests were applied by immersing the post-ultraviolet-cured specimens in a distilled water bath for 1-, 3-day, and 1-, 2-, and 4-week exposure times. The changes in flexural strain properties for each experimental parameter were also simulated by the computer-aided finite element analysis and compared with the experimental results. It was observed that the post-ultraviolet-curing process increased the flexural strength of the poly(lactic acid) specimens produced at both 190 and 200 °C with the same increasing trend up to 30 min of exposure, and the most significant increase was determined in the specimens that were subjected to post-ultraviolet-curing for 30 min. Although the flexural strengths of the post-ultraviolet-cured specimens were higher than the non-cured specimens in all conditions, it was detected that they tended to decrease after 30 min.

## 1. Introduction

**Figure 1:**
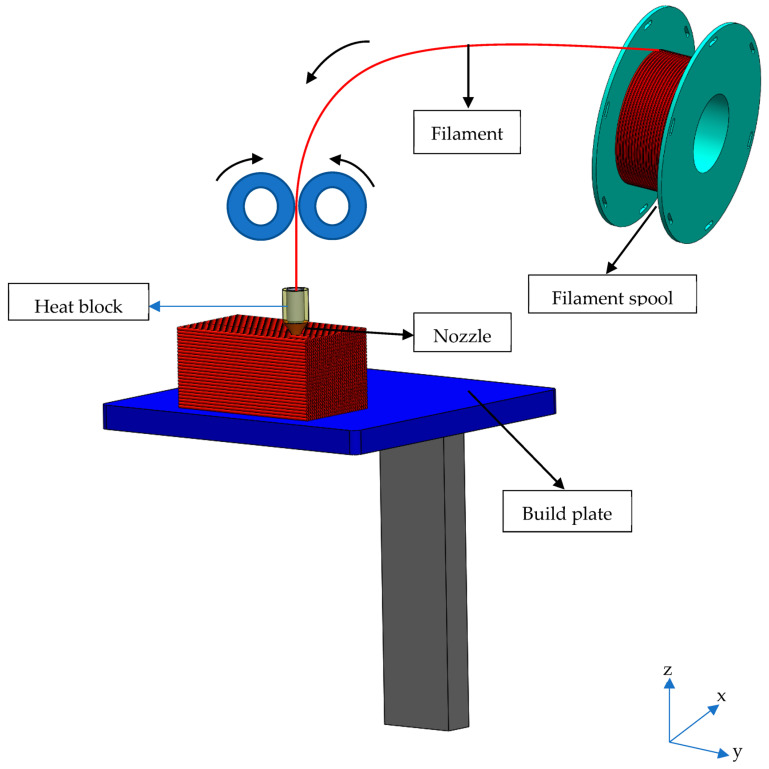
Schematic representation of a typical FDM type 3D printer.

Additive manufacturing (AM) technologies have become one of the competitive manufacturing techniques for the precise production of parts with high geometric complexity [1,2]. The most preferred AM method is fused deposition modelling (FDM), also referred to as fused filament fabrication (FFF) [3]. In FDM technology, the type of polymer used is liquefied and extruded in a semi-molten form using a nozzle with a particular effort and deposited on the path obtained by slicing the computer-aided design (CAD) geometry [4,5,6]. The part that reaches the final design with CAD software is converted into a stereolithography (STL) file and loaded into the software to divide the part into layers with cross-sections based on the specified layer thickness [3,7]. In this software, the G-code is obtained by determining the geometric parameters such as layer thickness, wall thickness, infill density and infill pattern, production parameters such as nozzle and build plate temperature, and print speed [8,9]. In this technique, the used filament is pushed towards the heated liquefier with the help of stepper motors and extruded using a nozzle as a semi-melt material. In order to deposit the semi-molten material on the determined path, the build plate or nozzle moves in the transverse and transitional directions. In this context, all paths on a layer are followed by completing the layer and passing it to the next layer [10]. A schematic representation of a typical FDM 3D printing technique is given in Figure 1.

FDM technology is a more accessible manufacturing technology than stereolithography (SLA) and selective laser sintering (SLS) in terms of equipment, cost, and materials used [11]. The fact that desktop 3D printers have an integrated system with open-source software and hardware provides complete control over parameters such as production speed and temperature. This ensures that a part produced using optimal geometric parameters will have better properties than those produced with expensive techniques [12]. The FDM technique, which produces one-piece limited-size geometries, is used in the aerospace, architectural, electronics, medical, and automotive industries for prototyping [13,14].

In the last decade, there has been an explosion in the number of desktop-type 3D printer users due to the diversification and ease of access of 1.75 and 2.85 mm diameter polymer filament consumables. The materials frequently used in the FDM method are poly(lactic acid) (PLA) [15,16,17], acrylonitrile butadiene styrene (ABS) [18,19,20], polyethylene terephthalate (PET) [21,22,23], polypropylene (PP) [24,25,26], polycarbonate (PC) [27], polyetheretherketone (PEEK) [28,29,30], thermoplastic polyurethane (TPU) [31,32,33], and polyamide (PA). In today’s market, apart from thermoplastics, it has become possible to reach varieties of composite materials such as glass fibre-reinforced PP [34], carbon fibre-reinforced PET [35], and carbon fibre-reinforced PA [36] and metallic materials such as stainless steel 316L [37] and 17-4ph [38].

PLA is the most preferred polymer filament in the FDM technique, which is used to obtain solid models with a 3D geometry by combining successive layers. Despite this variety of products, the reason why PLA, derived from agricultural products, is preferred first in most applications is its features such as biodegradability, not releasing toxic gases to the environment during processing, low melting temperature, low shrinkage level, and environmental compatibility [39,40]. Although PLA exhibits high mechanical properties, its application areas are limited due to some characteristic properties, such as low thermal resistance.

In order to eliminate material disadvantages, the researchers investigated how FDM technique production parameters and secondary post-production processes affect the mechanical, thermal, and other characteristics of PLA-based parts. Hsueh et al. [41] investigated the influence of FDM printing parameters on the characteristic behaviours of PLA and PETG. Production was carried out using temperatures in the range of 180–220 °C, and accordingly, it was observed that the tensile strength of PLA increased with increasing printing temperature. In another study, Hikmat et al. [42] investigated the effect of FDM production speed on the mechanical properties of PLA parts. Accordingly, PLA parts were produced with speeds of 20, 40, and 60 mm/s, and it was revealed that the tensile strength values of those produced with a speed of 20 mm/s were higher than the others. Rodríguez-Panes et al. [43] studied the effect of layer height, infill density, and layer orientation on the mechanical properties of PLA and ABS parts. Accordingly, as the infill density increased, the adhesion between the layers increased, and the porosity decreased with the increase in the contact area between the layers; therefore, an increase in the mechanical properties was observed. Hsueh et al. [44] fabricated PLA parts with the FDM technique using different printing temperatures (185 to 225 °C) and infill density ratios (10, 20, 33.3, and 50%) and then exposed the parts to 425 nm and 60 min of UV irradiation. According to the results, the tensile strength increased as the infill density and production temperature increased. UV irradiation decreased the tensile strength but increased Young’s modulus values in all samples.

When the literature was reviewed, it was noticed that many studies focused on the effect of FDM technique production parameters such as printing temperature, printing speed, layer thickness, and infill density on the characteristic properties of the produced material. On the other hand, very few studies investigate the changes in the properties of a material such as PLA, whose industrial importance and use have spread over a wide area, by applying secondary processes after production. In this study, 405 nm post-UV curing at different durations (15, 30, 45, and 60 min) was applied to PLA parts produced at different temperatures (190 and 200 °C) using the FDM technique, and changes in their flexural, absorptive, and morphological properties were observed by performing three-point bending tests, absorption tests, and scanning electron microscopy (SEM) analysis.

## 2. Materials, Manufacturing, and Specimen Preparation

CAD data of the test specimens were obtained using SolidWorks software. Files designed in SolidWorks were converted to STL file format and exported into Ultimaker Cura slicing software to obtain G-code. Industrial PLA filaments with a diameter of 1.75 mm, white colour and supplied by Ultrafuse (BASF 3D, Emmen, The Netherlands) were used. The properties of the PLA filament used in this study provided by the manufacturer are given in Table 1. Creality CR-05 Pro H (Creality 3D Technology Co., Ltd., Shenzhen, China) was used as the FDM-type 3D printer. Esun eBox (Esun Industrial Co., Ltd., Shenzhen, China) filament dryer was used at 40 °C to remove moisture from the filaments and improve the printing quality. The post-UV curing process was applied to the specimens using the Anycubic wash and cure 2.0 device (Anycubic Technology Co., Ltd., Hong Kong, China).

This study was mainly carried out to investigate the effects of production temperature and post-UV curing process on the flexural and absorptive properties of PLA parts produced by the FDM technique. In order to reduce the complexity of the study, only the production temperature and post-UV curing durations were chosen as parameters. According to the literature, as the production temperature of PLA with FDM, Andó et al. [45] used production temperatures varying between 190–220 °C, Hsueh et al. [44] chose production temperatures varying between 185–225 °C, Hsueh et al. [41] preferred production temperatures varying between 180–220 °C, Soares et al. [46] used production temperature as 200 °C, Alsoufi et al. [47] preferred temperatures ranging from 195–250 °C, and Valerga et al. [48] used temperatures ranging from 180–240 °C. Moreover, 190 and 200 °C were selected as the production temperature parameters, considering the fact that PLA production was carried out at the intervals mentioned in the research and the manufacturer’s production temperature range recommendation. Accordingly, considering the production parameters recommended by the manufacturer, 0.4 mm nozzle diameter, 60 °C bed temperature, and 50 mm/s production speed were chosen as fixed production factors. Rajpurohit et al. [49,50] and Tao et al. [51] preferred layer heights ranging from 0.1 to 0.3 mm, and Kamaal et al. [52] preferred 0.2 to 0.3 mm. Considering the layer height production parameter preferred by the researchers in their studies, 0.2 mm was preferred as the layer height. Samples produced using different raster angles are available in the literature [1,50,53]. Mostly raster angles of ±45° and 0–90° were used, so a raster angle of 45° was chosen as the fixed factor. In addition, Nida [53] also found that the PLA samples produced with a 45° raster angle were stronger than those with a 0–90° raster angle.

**Figure 2:**
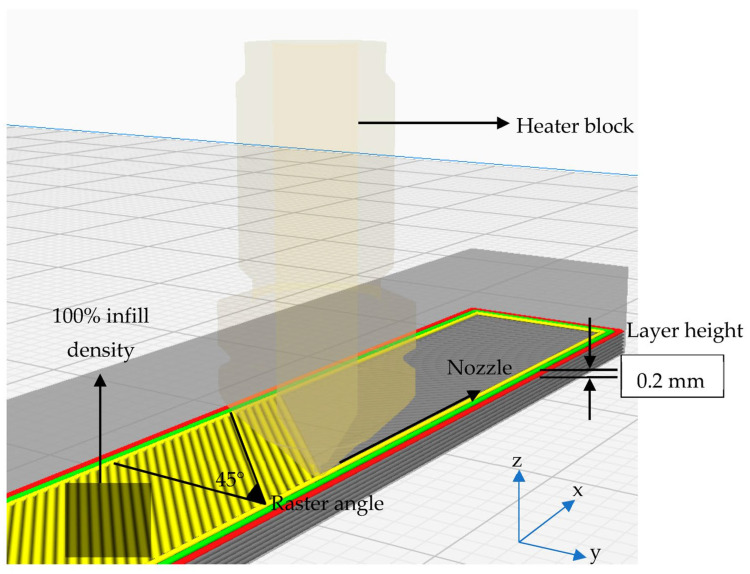
Schematic representation of the production parameters of the three-point bending specimen.

Specimens produced at 190 and 200 °C were subjected to post-UV curing at varying exposure times of 15, 30, 45 and 60 min. As the post-UV curing application, durations between 0 and 60 min at 15 min intervals were selected. Application durations of 0, 15, 30, 45, and 60 min were chosen since the curing device (Anycubic wash and cure 2.0 device) has a maximum continuous UV-curing application duration of 60 min, and the effect of different application durations on the characteristics of PLA has not been studied before. In addition to that, in the Hsueh study [44], only 60 min of UV-curing was applied to the samples produced with different parameters with the FDM technique. The printing parameters used in this study and the tags of the specimens are tabulated in Table 2 and Table 3, respectively. Schematic representation of the production process of the three-point bending specimen and X, Y, and Z labels for the build orientation are given in Figure 2.

### 2.1. Characterisation

#### 2.1.1. Flexural Test

**Figure 3:**
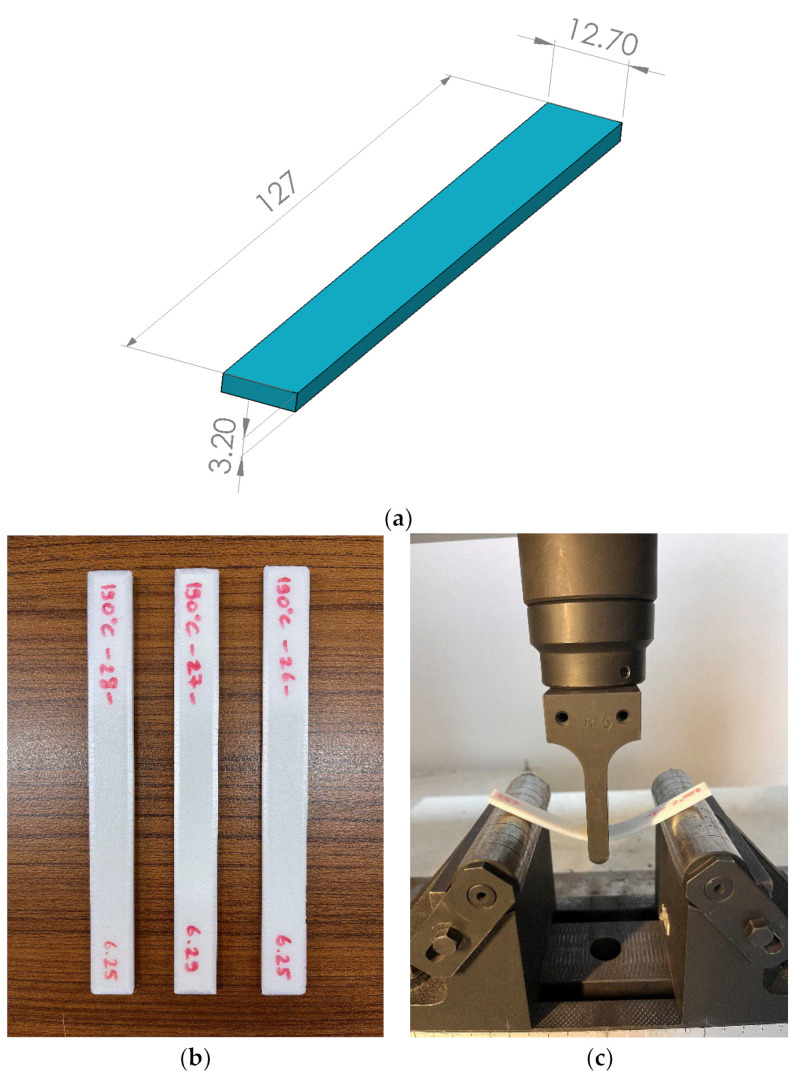
(**a**) CAD-model of the three-point bending test specimen according to ASTM D790 (Dimensions are given in millimetres), (**b**) produced test specimens, and (**c**) testing with Shimadzu 100 kN testing device.

Flexural characterisation of PLA parts exposed to 405 nm UV irradiation at different durations was performed by three-point bending tests. In order to minimise experimental errors, five tests were performed for each parameter, and the average values were recorded as test results. Three-point bending tests were performed according to ASTM D790 standard [54] to determine the behaviour of PLA parts against bending load. Three-point bending tests were carried out at 1 mm/min at room temperature using a Shimadzu 100 kN device. As a result of these tests, bending properties such as flexural strength and flexural modulus were determined. Dimensions of a three-point bending test specimen, produced specimens, and during testing with Shimadzu 100 kN instrument are given in Figure 3.

Calculation of flexural strength ($\sigma_{f}$), the maximum flexural strain ($\varepsilon_{f}$), and flexural modulus ($E_{f}$) are obtained using the following equations in accordance with ASTM D790 standard [54] based on classical beam theory. From the data, flexural strength ($\sigma_{f}$) is calculated assuming that the shear stress effects are in the negligible direction since the sample has a sufficient span-thickness ratio. The flexural strength ($\sigma_{f}$) in the three-point bending test is defined as

(1) $$\sigma_{f}=\frac{3PL}{2bd^{2}}$$

where P is the load at a given point on the load-deflection curve, L is the support span distance, b is the width of the specimen, and d is the thickness of the specimen.

The flexural strain ($\varepsilon_{f}$) value is calculated as follows

(2) $$\varepsilon_{f}=\frac{6Dd}{L^{2}}$$

where the deflection in the mid-span is defined by D.

**Figure 4:**
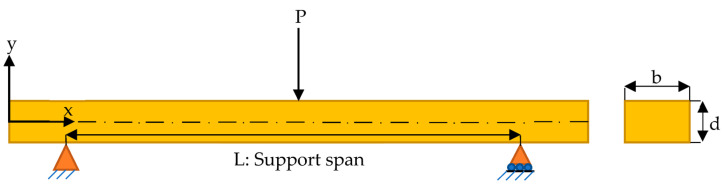
Simply supported beam with a concentrated load from the centre of the support span.

Flexural modulus ($E_{f}$) represents the ratio of stress to corresponding strain value at any point in the linear portion of the flexural strength–strain curve and is expressed by the following Equation

(3) $$E_{f}=\frac{L^{3}m}{4bd^{3}}$$

where m defines the tangent to the initial line portion of the load–displacement curve. A schematic illustration of a simply supported beam being loaded with a concentrated load from the centre of the support span is given in Figure 4.

#### 2.1.2. Absorptive Test

Absorption samples produced according to ASTM D570 [55] standard were dried at 60 °C for 5 h after production and allowed to cool at room temperature. Before undergoing any treatment, the initial weights of the samples were measured using a 1/10,000 precision balance (Radwag AS 220/C/2). They were immersed in a distilled water bath at room temperature for 1 day to 4 weeks at different water absorption times. After the exposure, the samples were dried with a dry cloth and kept at room temperature for 1 day. Water gain percentages were calculated according to Equation (4).

(4) $$\mathrm{Percent}\;\mathrm{Water}\;\mathrm{Absorption}\;(\%)=\frac{\mathrm{Wet}\;\mathrm{weight}\;-\;\mathrm{Dry}\;\mathrm{weight}}{\mathrm{Dry}\;\mathrm{weight}}\times 100$$

#### 2.1.3. Morphological Properties

The morphology of the PLA parts was analysed using SEM analysis. In order to obtain surface micrographs, field emission scanning electron microscope Carl Zeiss 300VP device with 15 kV acceleration voltage was used in accordance with the ASTM E986 standard [56]. Before examining the surface morphology, the specimens were coated with 5 nm gold vanadium. The plating process was carried out in 120 s under a vacuum with the ION COATER COX EM brand gold plating device.

#### 2.1.4. Finite Element Analysis and Modelling

The computational modelling procedure of the simply supported beams with a concentrated load from the centre of the support span was explained in this section. In the finite element analysis (FEA), the three-point bending specimen CAD model (Figure 3a) designed for experimental production was transferred to the ANSYS Workbench 2022 R1 simulation program. For computational modelling, the object geometry was designed using SolidWorks software and then imported into the Ansys Workbench SpaceClaim geometry module. In the mesh module, the object is divided into 624 elements and 3901 nodes using the 3D higher-order SOLID186 element type with 20 nodes and 3 degrees of freedom (3-DOF), which is used to model the behaviour of materials and structures such as deflection, plasticity, and hyperelasticity. FEA’s constraints and boundary conditions were identified similarly to the experimental test setup.

## 3. Results and Discussion

### 3.1. Experimental Results

**Figure 5:**
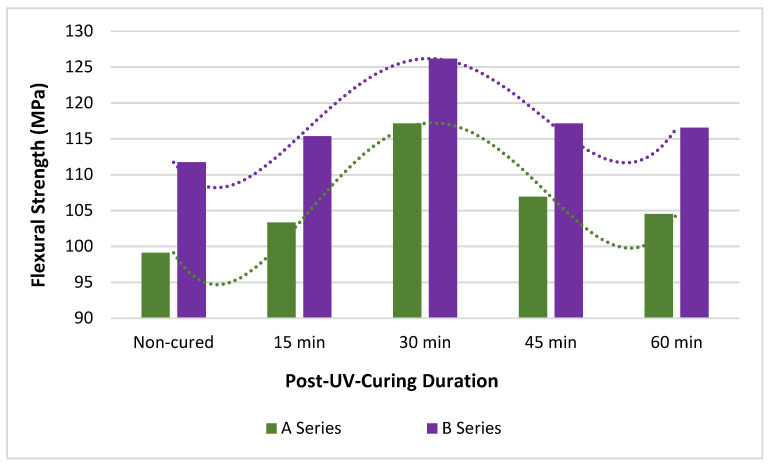
Flexural strength—post-UV curing duration graph of PLA parts.

**Figure 6:**
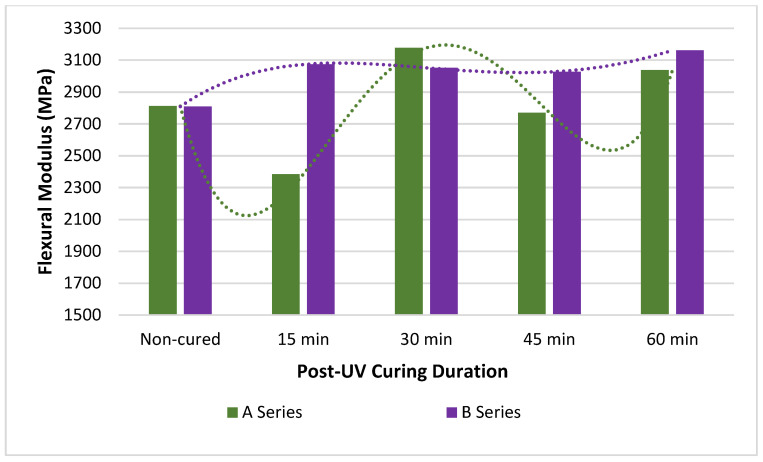
Flexural modulus—post-UV curing duration graph of PLA parts.

The flexural strength and modulus values with trend lines of post-UV-cured PLA specimens for different durations are given in Figure 5 and Figure 6, respectively. In addition, flexural strength and modulus values are given in Table 4 with standard deviations. In Table 4, the up-down trends of the samples compared with the non-cured specimen data are indicated with arrows and the ratio of the percentage change. When evaluated in terms of flexural strength values, it is observed that A3 and B3 samples have the highest value, and A1 and B1 have the lowest value regardless of the production temperature. When Table 4a is examined, it is seen that the A3 increased by 18.19% compared to the A1, and the B3 increased by 12.91% compared to the B1. When A1 and B1 are taken as references, it is determined that the lowest increase is in A2 (4.25%) and B2 (3.22%), respectively. When evaluated in terms of flexural strength, non-cured samples produced at 190 °C show lower values than those produced at 200 °C, and this relative situation maintains the trend after each UV irradiation, thus; B1 shows lower values than A1, B2 than A2, B3 than A3, B4 than A4, and B5 than A5.

PLA shows lower mechanical strength at high temperatures than the polymer frequently used in FDM technology, such as ABS, but this is preferable at ambient temperatures [11,57,58,59]. A part made of ABS polymer may fail due to delamination under a minimum load, owing to residual stresses arising from the hindered shrinkage of the polymer during the cooling process. The parts produced using PLA, which has a lower shrinkage rate, turn into a product with less internal stress and exhibit better mechanical properties. The high performance of the 3D printed parts depends on the strength and stability of the bonds formed between the layers of the sample, which are usually determined by the printing parameters, except for the characteristic properties of the filament material [11]. Printing temperature, one of FDM techniques’ primary and most effective production parameters, affects the rheological properties, crystallinity, deformation, and thermal and mechanical properties of polymeric filament sections. It affects the bond strength between the layers and the lines, causing a change in the mechanical properties; in addition, the printing temperature parameter influences FDM printability and the macro mechanical properties of the printed part [59,60,61]. Production with low printing temperatures causes the polymer to melt with low fluidity and high viscosity. This results in the formation of a large amount of porosity between the layers and lines of the molten PLA. The porous structure leads to a reduced contact area and poor bond formation between layers and lines; thus, the production of materials with low values in terms of mechanical properties occurs [41,48,62]. Especially when the flexural strength values of A1 and B1 samples are compared with each other, the higher flexural strength of B1 samples produced by preferring higher production temperature is due to the higher bonding success between rasters. Since the production temperature is directly related to the degree of crystallinity, the decrease in the mechanical properties of the samples produced with very high production temperatures can be explained by the deterioration of the molecular chain and the gradual collapse of the structural layers [63,64,65,66].

**Figure 7:**
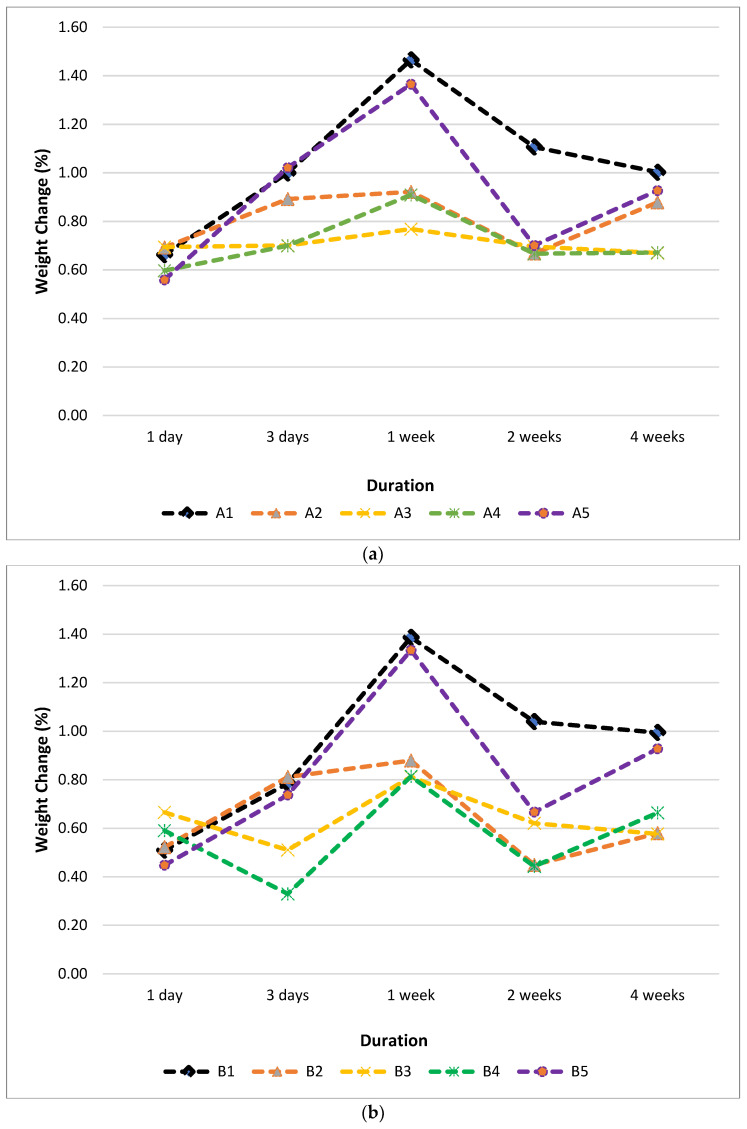
Weight change—duration graphs of post-UV-cured and produced at (**a**) 190 °C (**b**) 200 °C PLA parts.

**Figure 8:**
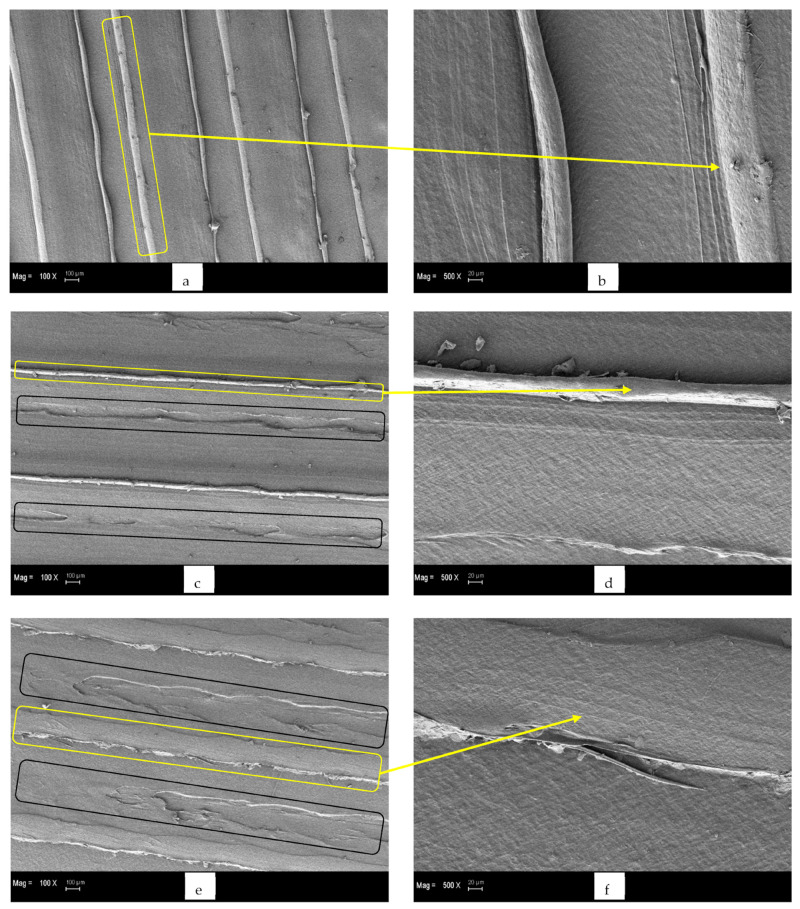
SEM micrographs of (**a**,**b**) non-cured, (**c**,**d**) 30 min post-UV-cured and (**e**,**f**) 60 min post-UV-cured PLA specimens produced at 190 °C.

Weight change—duration water absorption behaviour graphs of PLA samples are given in Figure 7. After 1 week of exposure, all samples show the highest absorptive ability. It is observed that the absorption amount is at the highest level among all A and B series samples, which were kept in distilled water for a week. It is determined that the 60 min post-UV-cured samples generally have the highest absorption capacity for all periods. The absorptive ability of non-cured samples is at the lowest level for all samples and all durations. Thanks to the porous structure arising from the nature of production with the FDM technique, water uptake can be achieved towards the inner layers of the samples. Although the samples with the post-UV curing process show different water uptake values compared to the non-cured samples, the trend shows the same direction and approximately similar behaviour. Its water intake capability causes reduced pores when production is made with high production temperature, and therefore less water intake can occur [14,67]. The sample water intake capabilities have the highest value among themselves due to the irregularities and deteriorations on the A5 and B5 sample surfaces (Figure 8e,f).

**Figure 9:**
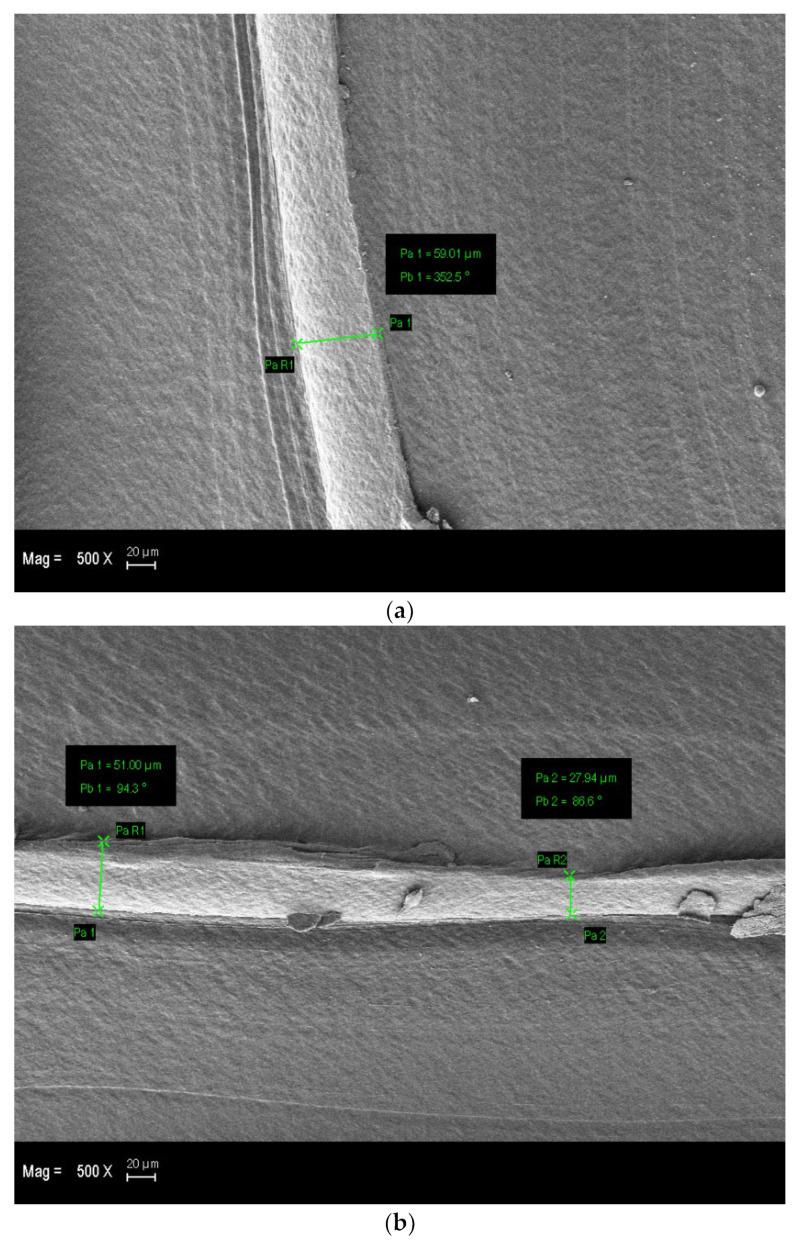
Diameter measurement for the printing fibres of (**a**) non-cured and (**b**) 30 min post-UV-cured PLA specimens produced at 190 °C.

SEM micrographs of non-cured (a, b), 30 min post-UV-cured (c, d), and 60 min post-UV-cured (e, f) samples produced at 190 °C are given in Figure 8. Printing lines are indicated with yellow frames, and surface deformations are indicated with black frames on the SEM micrographs. Accordingly, the printing lines, which are distinctly prominent and regular in the non-cured samples (Figure 8a,b) and samples exposed to UV irradiation for 30 min (Figure 8c,d), turn into discontinuous, irregular, non-uniform, and less obvious printing lines on the PLA micrograph exposed to UV light for 60 min (Figure 8e,f). In addition, the measured diameters of printing fibres of (a) non-cured and (b) 30 min post-UV-cured samples in the SEM micrographs are given in Figure 9. Accordingly, the diameter of the printing fibres on the non-cured specimen’s outer surface is larger than that of the specimens that were post-UV cured for 30 min. The post-UV curing process reduces the prominence of the fibres on the sample surface and causes their diameter to decrease. This may be due to the fact that the post-UV curing process causes the rearrangement of molecular chains on the material surface to be tighter and the distance between molecular chains shortened [44,68]. Correspondingly, the reason why A3 and B3 samples have the highest flexural strength values in the A and B series samples may be that the UV-irradiation process forms a stronger adhesion combination between the printing fibres and reduces the number of air gaps on the surface and in the internal structure of the PLA specimens [69]. Surface degradations that are not encountered in the non-cured samples but that occur in the samples exposed to UV irradiation for 30 and 60 min are also clearly visible, and it has been noticed that the surface area of these craters increases with the increase in the UV-irradiation time. The decrease in flexural strength values after 30 min with the increase in the UV-irradiation exposure time may have resulted from the increase in the area of the deformation zones and surface irregularities on the material surface, as can be seen in Figure 8. The surface degradation and irregularities seen in the PLA samples, especially after more than 30 min of post-UV curing, may be due to the ageing phenomenon of the post-UV curing process. As a result, the relatively unequal bond strength between the surface fibres of PLA has occurred, and the bonding success between printing fibres has decreased. The fact that PLA has a biodegradable structure, the ageing phenomenon triggers this process, causing the material to begin degrading. The degradation of the material by such an accelerated effect may be due to the active material degradation mechanism and the occurrence of chain breaks [70,71,72].

### 3.2. Finite Element Analysis Results

**Figure 10:**
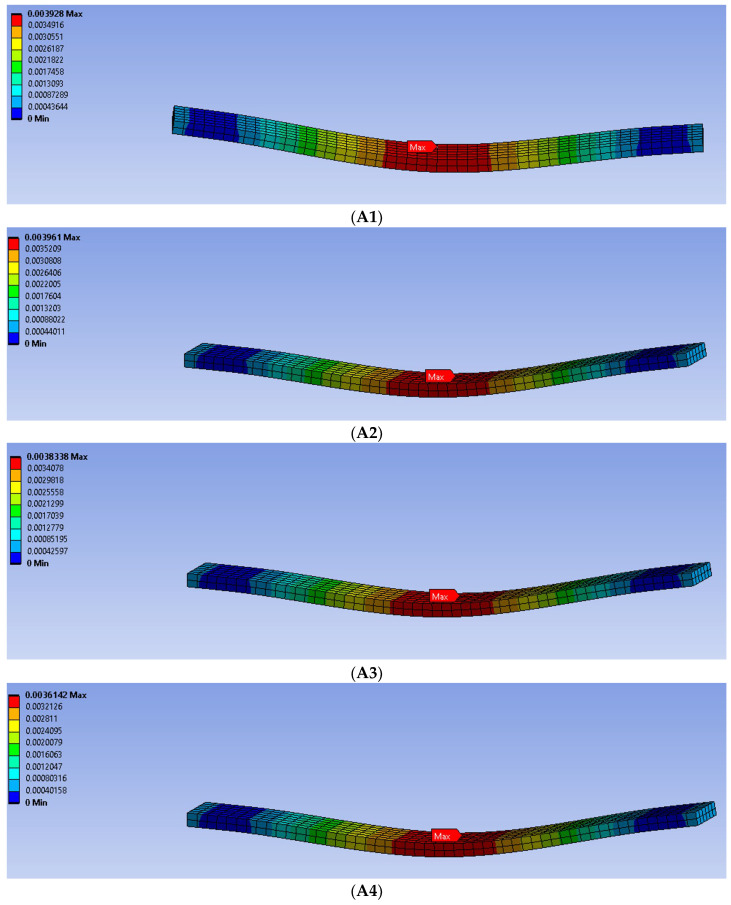

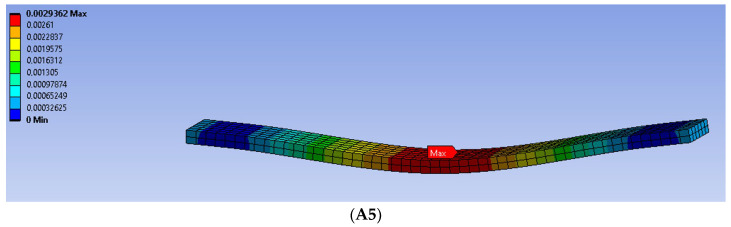
Deflections of the (**A1**–**A5**) PLA beams.

The deflection results of the specimens subjected to a three-point bending test were obtained with the FEA. Figure 10 shows contour graphs of displacement results obtained with ANSYS Workbench. The experimental results were compared with the FEA results attained using ANSYS Workbench. The average error in deflection (δ) was calculated as follows:

(5) $$\mathrm{error}\;(\%)\;\delta =\frac{\left|\delta_{FEA}-\delta_{\exp eriment}\right|}{\delta_{\exp eriment}}\times 100\%$$

In order to get the deflection values from the FE calculations, the elements located in the middle sections of the support span of the beams were used. Table 5 shows the average deflection error percentages obtained using Equation (5). Comparisons were made by finding the deflections corresponding to a specified constant force value in the linear part of the load–displacement curve of each sample. In the study of Abouelmajd et al. [73], they stated that the average error between the flexural strength values found between the experimental and FEA analysis was at the level of 5%. Gebrehiwot et al. [74], on the other hand, stated that the average error between the numerical and experimental results of the deflection and strength values of PLA flexural samples produced by the FDM technique was below 10%. Accordingly, it is seen that the results of the FEA are in good agreement with the experimental results.

## 4. Conclusions

This study examines how the post-UV curing process at different exposure times affects the flexural, absorptive, and morphological properties of PLA specimens. The findings obtained as a result of experimental studies are itemised below.

- Increasing the printing temperature causes the material to have better flexural strength. The high printing temperature causes the flexural strength values of B1 samples to be 12.73% higher than A1 specimens, B2 to be 11.62% higher than A2, B3 to be 7.69% higher than A3, B4 to be 9.56% higher than A4, and B5 to be 11.50% higher than A5.
- When the UV-irradiation application is examined for the flexural strength values of the samples, cured samples show higher values than non-cured samples, regardless of the temperature at which they are produced. The application of the post-UV curing process causes the flexural strength values of the samples to behave characteristically the same regardless of the A and B series. Up to 30 min of post-UV curing application causes the flexural strength values to increase gradually, reaching the highest value after 30 min and hovering around a certain value after 30 min of application. Among both A and B series specimens, 30 min post-UV-cured ones have the highest flexural strength and non-cured ones have the lowest flexural strength. When A series specimens are compared with each other in terms of flexural strength, A2 is 4.25% higher than A1, A3 is 13.37% higher than A2; however, A4 is 8.72% lower than A3, and A5 is 2.24% lower than A4. When this comparison is made for the B series specimens, B2 is 3.22% higher than B1, B3 9.38% higher than B2, although B4 is 7.14% lower than B3, and A5 0.51% lower than A4.
- When the deflection values formed in the samples with three-point bending tests are examined experimentally and numerically, the highest deviation between the experimental and FEA results is seen in the B3 sample with 3.28% and the lowest value in the A5 and B5 samples with 1.15%. When all deviation values are examined, it can be said that there is a good agreement between experimental and numerical analysis.

## Figures and Tables

**Table 1:** Material properties and suggested printing parameters by the manufacturer.

Material Properties	Unit	Value
Density	(kg/m^3^)	1248
Nozzle temperature	(°C)	190–230
Bed temperature	(°C)	50–70
Melting temperature	(°C)	151
Nozzle diameter	(mm)	≥0.4
Printing speed	(mm/s)	40–80

**Table 2:** Production parameters.

Production Parameters	Unit	Value
Moulding technology	-	FDM
Layer height	(mm)	0.2
Top and bottom thickness	(mm)	0.8
Infill density	(%)	100
Print material	-	PLA
Filament diameter	(mm)	1.75
Bed temperature	(°C)	60
Nozzle temperature	(°C)	190, 200
Printing speed	(mm/s)	50
Raster angle	(°)	45

**Table 3:** Specimen details.

Post-UV-Curing Duration (min)		0	15	30	45	60
**Printing Temperature (°C)**	190	A1	A2	A3	A4	A5
	200	B1	B2	B3	B4	B5

**Table 4:** (**a**) Flexural strength and (**b**) flexural modulus of PLA parts exposed to post-UV curing.

**(a)**		**Flexural Strength (MPa)**							
**190 °C**					**200 °C**				
**Non-Cured**		**Duration** **(min)**	**Code**	**Cured**	**Non-Cured**		**Duration** **(min)**	**Code**	**Cured**
A1	99.12 (±3.61)	15	A2	103.33 (±0.81) 4.25%	B1	111.74 (±0.52)	15	B2	115.34 (±0.65) 3.22%
		30	A3	117.15 (±1.85) 18.19%			30	B3	126.16 (±1.68) 12.91%
		45	A4	106.93 (±2.52) 7.88%			45	B4	117.15 (±2.52) 4.84%
		60	A5	104.53 (±3.52) 5.46%			60	B5	116.55 (±2.05) 4.30%
**(b)**		**Flexural Modulus (MPa)**							
**190 °C**					**200 °C**				
**Non-Cured**		**Duration** **(min)**	**Code**	**Cured**	**Non-Cured**		**Duration** **(min)**	**Code**	**Cured**
A1	2812.35 (±3.61)	15	A2	2385.90 (±25.61) −15.16%	B1	2808.77 (±0.52)	15	B2	3073.14 (±65.46) 9.41%
		30	A3	3177.42 (±126.91) 12.98%			30	B3	3052.52 (±96.38) 8.68%
		45	A4	2770.03 (±32.68) −1.50%			45	B4	3026.73 (±25.13) 7.76%
		60	A5	3037.64 (±38.41) 8.01%			60	B5	3162.03 (±38.43) 12.58%

**Table 5:** The deviation between experimental and FE analysis results.

Specimen	Average Deflection Error (%)
**A1**	%1.26
**A2**	%2.38
**A3**	%2.21
**A4**	%3.01
**A5**	%1.15
**B1**	%1.48
**B2**	%1.35
**B3**	%3.28
**B4**	%2.21
**B5**	%1.15

## Data Availability

The data presented in this study are available on request from the corresponding author.
